# ‘Bern, get ready’, BEready, a household-based cohort study for pandemic preparedness research in Switzerland: pilot study

**DOI:** 10.1136/bmjopen-2025-109555

**Published:** 2025-12-07

**Authors:** Eva Maria Hodel, Selina Wegmüller, Franziska Iff, Emily Lim, Karin Grimm, Lea Gasser, Laura Di Domenico, Simone Schuller, Gilles Wandeler, Nicola Low

**Affiliations:** 1 Multidisciplinary Center for Infectious Diseases, University of Bern, Bern, Switzerland; 2 Institute of Social and Preventive Medicine, University of Bern, Bern, Switzerland; 3 Department of Clinical Research, University of Bern, Bern, Switzerland; 4 Institute of Philosophy, University of Bern, Bern, Switzerland; 5 Department of Infectious Diseases, Inselspital University Hospital Bern, Bern, Switzerland; 6 Department of Clinical Veterinary Medicine, University of Bern, Bern, Switzerland; 7 Data Science Institute, Hasselt University, Hasselt, Belgium

**Keywords:** INFECTIOUS DISEASES, Epidemiology, Epidemics, Observational Study

## Abstract

**Abstract:**

**Objectives:**

Research for pandemic response needs to be timely to inform evidence-based decision making. The lack of epidemiological data at the start of the COVID-19 pandemic led experts to call for cohorts that could rapidly supply data about newly emerging infectious diseases. The ‘Bern, get ready’ (BEready) study aims to establish a prospective ‘pandemic preparedness cohort’ in the canton of Bern, Switzerland. This cohort can be pivoted to the needs of a new pandemic pathogen. The aim of this pilot study was to investigate the potential response and to test the feasibility of procedures for BEready.

**Design:**

Closed population-based cohort study.

**Setting:**

Random sample of private households in the canton of Bern, Switzerland, that had previously responded to an online survey.

**Participants:**

Adults, children and pets.

**Primary and secondary outcome measures:**

Enrolment as a percentage, associations between the agreement to participate and the demographic and socioeconomic variables of the invited household member, number of social contacts, proportion of samples collected, proportion of complete questionnaires and proportion of participants responding after 12 months.

**Intervention:**

After the initial in-person visit with venous blood sampling, participants were followed up for 1 year. We tested remote data collection methods, with online questionnaires and self-collected capillary blood and nasopharyngeal samples, and established a biobank.

**Results:**

The pilot study enrolled 106/1138 (9%) of invited households plus two additional households that had proactively contacted us. In total, we enrolled 193 people in 108 households (1.8 per household) and 44 pets between April and September 2023. We obtained and stored at least one venous and/or capillary baseline blood sample from 184/193 (95%) people and 40/44 (91%) pets. After 1 year, 172/193 (89%) people in 101/108 (94%) of households completed a follow-up survey, as did 22 owners of 34/44 (77%) pets. 151/172 (88%) respondents returned a follow-up capillary blood sample.

**Conclusions:**

The response rate to the pilot study shows that obtaining high levels of participant enrolment in a pandemic preparedness cohort study is challenging. Data collection without face-to-face contact with a study team is feasible for household members and will be needed in BEready if control measures during a pandemic prevent in-person studies.

**STRENGTHS AND LIMITATIONS OF THIS STUDY**

- This pilot study was designed to test all procedures planned for the main cohort study, including the invitation process, the in-person visit at enrolment, and the data and sample collection methods.
- Due to its small sample size, the study had low statistical power to assess factors influencing participation.
- Online questionnaires and mailed self-collected samples were efficient data and sample collection methods that minimised direct contact with study staff, although self-collection limited the types and volumes of samples obtained.
- Limiting participation to individuals with sufficient German or French language skills and internet access excluded certain groups of the population.
- Focusing solely on cats and dogs covered the most common household pets but limited range of animal-human infections which can be studied.

## Introduction

 Research for pandemic response needs to be timely. In Switzerland, the first seroprevalence data were publicly available only 3 months after the first case of COVID-19 was confirmed on 25 February.^1 2^ These much-needed data came from an established population-based study, Bus Santé,^3^ which collected information on cardiovascular risk factors in the canton of Geneva. The seroprevalence study showed that, by the end of the first wave of COVID-19 in Geneva, only about 10% of study participants had developed detectable antibodies to the virus. This indicated that most of the population of Switzerland had remained unexposed and potentially immunologically unprotected against future waves of COVID-19.^2^ Other countries faced similar challenges and, in 2020, the Global Research Collaboration for Infectious Disease Preparedness called for ‘pandemic preparedness cohorts’. These cohorts would establish research infrastructure and community engagement and be able to ‘pivot’ rapidly at the start of a new pandemic.^4^

Active longitudinal cohort studies are valuable in pandemic research because they collect rich clinical and laboratory data over time, enabling deeper insight, for instance, into effect modifiers of disease outcomes.^4^ To our knowledge, none of the existing large population-based longitudinal cohorts has been designed to specifically collect longitudinal data relevant for pandemic preparedness. The ‘Bern, get ready’ (BEready) study aims to establish a prospective ‘pandemic preparedness cohort’ in the canton of Bern, Switzerland. BEready will be a population-based cohort study, with infrastructure to conduct research about infectious diseases when there is no public health emergency and to respond to the needs of a new pandemic pathogen. Infections typically cluster within households due to shared environmental and behavioural exposures and the potential for intra-household transmission of pathogens which spread through direct contact. Households, therefore, are the unit of enrolment to allow the study of both primary and secondary infection events, to facilitate the collection of shared exposure data, and to reduce bias arising from treating related individuals as independent units. A population-based cohort can collect data about infection-related issues, including social mixing, which are essential for mathematical modelling of the transmission and potential control measures for infectious diseases.^5^ Data about pre-pandemic social mixing patterns were absent in Switzerland at the start of the COVID-19 pandemic, which meant that data from other countries or from synthetically constructed matrices needed to be used.^6^ Furthermore, two-thirds of emerging and re-emerging infectious diseases are of animal origin.^7^ Including household pets will help to study infections at the human-animal-environmental interface, which aligns with the One Health approach.^8^

In autumn 2022, we explored the willingness of people in the canton of Bern to participate in a future research study focusing on infectious diseases in an online survey.^9^ Of 15 000 randomly selected adults living in a private household, 23% responded and about half of these were willing or quite willing. The objective of this study was to investigate the expected participation rate and to test the feasibility of procedures for a population-based household cohort study for pandemic preparedness research in the canton of Bern.

## Materials and methods

We conducted a closed cohort study, with a baseline visit and 1 year of follow-up. We tested methods which allow remote data and sample collection without face-to-face contact with a study team because control measures during a pandemic might prevent in-person investigations. We report the study using the Strengthening Reporting of Observational Epidemiological Study checklist for cohort studies.^10^

### Study setting and participants

The canton of Bern is the second largest canton in Switzerland geographically, with demographic characteristics similar to those of the whole country,^11^ and in which the number of reported COVID-19 infections and associated mortality were average for Switzerland.^12^ In autumn 2022, we invited a random sample of respondents to the online survey^9^ who had given permission to be contacted again (online supplemental material). The project management team at the Institute of Social and Preventive Medicine, University of Bern, sent an invitation letter, by surface mail, which explained the purpose of the study and contained a quick response code with a link to a registration form. We sent one reminder letter around 2 weeks after the invitation. We also invited potentially eligible households who had contacted us and shown interest in participating in the study. We did not conduct a formal sample size calculation because this was a pilot study, without hypotheses. The goal was to test procedures and we judged 100 households to be sufficient.

In this pilot study, each household nominated a main contact person, typically the person who received the invitation letter. The main contact person registered the household online using Research Electronic Data Capture (REDCap) tools hosted at the University of Bern.^13 14^ Eligibility criteria were: half, or more than half, of household members (including at least one member aged 18 or above) agreed to take part; the main household contact lived (at least 4 days/week) in a private household in the canton of Bern, had access to email and internet, sufficient oral and written knowledge of German and/or French and intended to remain in the study area for the duration of follow-up. We asked them to enrol as a household, including household pets (cats and dogs only). We did not offer incentives to human participants but, to encourage enrolment of pets, we offered pet owners a pet health check and blood tests.

### Study procedures

The project management team telephoned the main contact person to arrange a video conference for the household. They sent an email containing a link to written information about the study, which was available in age-appropriate formats for adults and children aged 14+ years, children aged 11–13 years, children up to 10 years, toddlers, parents/legal guardians and pet owners. During the video call, a study nurse or research assistant checked the eligibility criteria, explained the study and answered questions. If half, or more than half, of the household members still agreed to participate, the study nurse or research assistant arranged a baseline visit either at the study centre (Swiss Institute for Translational and Entrepreneurial Medicine, Bern) or at home, according to participants’ preferences. If fewer than half of the household members agreed to participate, the study nurse did not offer a baseline visit for enrolment. In this pilot study, we aimed to test procedures for data and sample collection among participants experiencing symptoms of respiratory infections in households with at least two people.

At the baseline visit, a study nurse first asked the participants to sign, confirming their informed consent. The study nurse measured height, weight, hip and waist circumference, pulse and blood pressure and took 15 mL venous blood and 5 drops of capillary blood on filter paper from each participant (online supplemental table S1). The study nurse showed participants how to take a nasal swab from themself and their children and how to collect a capillary blood sample on filter paper (dried blood spots).

After the baseline visit, participants filled in an online questionnaire using REDCap. For children under the age of 14 years and pets, an adult in the household filled in the questionnaire. Where possible, we used existing questionnaires, which were part of national and multinational studies (online supplemental table S2). The questionnaire covered sociodemographic characteristics, human-animal contact, quality of life, medical history, vaccinations and travel history. The Swiss neighbourhood index of socioeconomic position^15^ was determined based on the household’s residential address. We asked participants to complete a social contact survey, reporting on the number of contacts during a 24-hour period, starting at 05:00 on the previous day.^16^

A study veterinarian conducted the baseline visit for cats and dogs at the small animal clinic, Vetsuisse Faculty, University of Bern, including a medical history and physical examination. The clinical examination included an estimation of the Body Condition Score to assess nutritional status and body fat of an animal.^17 18^ A study veterinarian or nurse took venous blood from pets and separated it into samples of plasma and serum (online supplemental table S1). The study veterinarian showed participants how to take a nasal swab from dogs or a pharyngeal swab from cats (online supplemental table S1). The central laboratory of the Vetsuisse Faculty Bern performed biochemistry and haematology analyses, and the study veterinarian gave the pet owners the results by telephone and via email.

A study nurse or the study veterinarian transported biological samples to the Liquid Biobank Bern, Switzerland.^19^ Biobank staff generated aliquots and stored them at −80°C (online supplemental table S1).

After 1 month, the project management team emailed the main household contact and asked them to complete a REDCap online questionnaire about satisfaction with the study procedures. Six weeks before the end of the 1 year follow-up, they sent a sampling kit to the household contact, asking each participant to collect five drops of capillary blood on filter paper from themselves and children under 14 years old and send the dried blood spots by prepaid surface mail to the biobank for storage. Four weeks before the end of the 1 year follow-up, participants received an automated invitation to fill in a follow-up questionnaire online. An adult filled in the questionnaire for children under 14 years and pets. The questionnaire covered any changes since the baseline visits (online supplemental table S2). Efforts were made to follow-up on participants who had not completed the questionnaire (online supplemental material).

Throughout the study period, we asked participants to report, online in REDCap, if they experienced any of four symptoms lasting more than 24 hours: cough, runny nose, sore throat and/or shortness of breath. The study physician contacted each person reporting symptoms for a telemedicine consultation and instructed them to self-collect a nasal swab and, after a week, for all other household members, including asymptomatic people and pets, to take a nasal or pharyngeal swab. The households sent the swabs by prepaid surface mail in universal transport medium to the biobank (online supplemental table S1). A laboratory technician tested swabs in batches for respiratory viruses (online supplemental material). These results will be reported separately.

### Statistical analyses

We performed all analyses in the statistical package R.^20^ The primary outcome was enrolment as a percentage, which we calculated by dividing the number of households enrolled by the number of households invited for the pilot study. We excluded households who proactively volunteered to take part from the denominator for the response rate.

Secondary outcomes included the associations between the agreement to participate and the demographic and socioeconomic variables of the invited household member, the number of social contacts, the proportion of participants from whom samples were collected, the proportion of participants with completed questionnaires and the proportion of participants responding after 12 months.

We described household characteristics as frequencies (and percentage) or medians (with IQR). We also explored associations between the agreement to participate and the demographic and socioeconomic variables of the invited household member. For some variables (nationality, level of education, location of residence and household income), we combined the categories into fewer groups. The unit of analysis was the person receiving the invitation letter. For households that did not accept the invitation to participate in the pilot study, we only had demographic and socioeconomic information for the invited person, not for other household members. We fitted univariable logistic regression models using the glm() function with a logit link function and expressed associations between participation and demographic characteristics as OR with 95% CIs. We excluded observations with missing values. We performed multivariable analyses to adjust for potential confounders and expressed the results as adjusted ORs (aOR, with 95% CI). Potential confounders (age, sex, nationality, education, household size, urban-rural typology and income) were selected a priori. We selected age, education, household size, urban-rural typology and income as potential confounders based on the analysis of our online survey.^9^ The online survey showed that willingness to participate was less likely among older participants and those in larger households, and more likely among participants with the highest educational level, near the city of Bern than in rural areas, and those with the highest income. The additional confounders, sex and nationality, were selected based on subject matter knowledge. We assessed the contribution of each demographic and socioeconomic characteristic to the odds of participation using likelihood ratio tests, comparing the full multivariable model with models excluding that variable. We assessed multicollinearity by calculating generalised variance inflation factors (VIFs) using the VIF() function in the ‘regclass’ package.

We fitted linear mixed-effects models using the ‘lme4‘ package, with a random intercept per household, to estimate the average number of days for each person or pet from inclusion to the day of last contact. We analysed the number of social contacts and constructed a social contact matrix using methods previously described (online supplemental material).^56 21^,^24^ We used the estimated contact matrix to quantify the variation in the reproductive number R0 compared with pre-pandemic contacts. Details on the construction of the contact matrix and derivation of R0 are reported in the supplementary material.

### Patient and public involvement

The BEready cohort has a strong emphasis on community engagement and aims to engage diverse communities in ongoing efforts to strengthen pandemic and public health literacy, and to prepare for the public health response to future pandemics. Findings from two sources informed the design of this pilot study. In autumn 2022, we explored the willingness of people in the canton of Bern to participate in a future research study focusing on infectious diseases in an online survey.^9^ We then held two stakeholder meetings in May 2022 and February 2023 with representatives of the cantonal and federal public administration, professional associations and think tanks, where we received feedback on the design and conduct of the pilot study. During the set-up of the pilot study, we founded our community engagement committee, which has been growing since. It involves interested people of the public, cohort participants and people with experience in engaging with patients and the public. The community engagement committee will continuously provide input in, among others, the design of the main study, especially the enrolment strategy, as well as our dissemination and engagement activities at public events.

## Results

### Participant numbers

**Figure 1:**
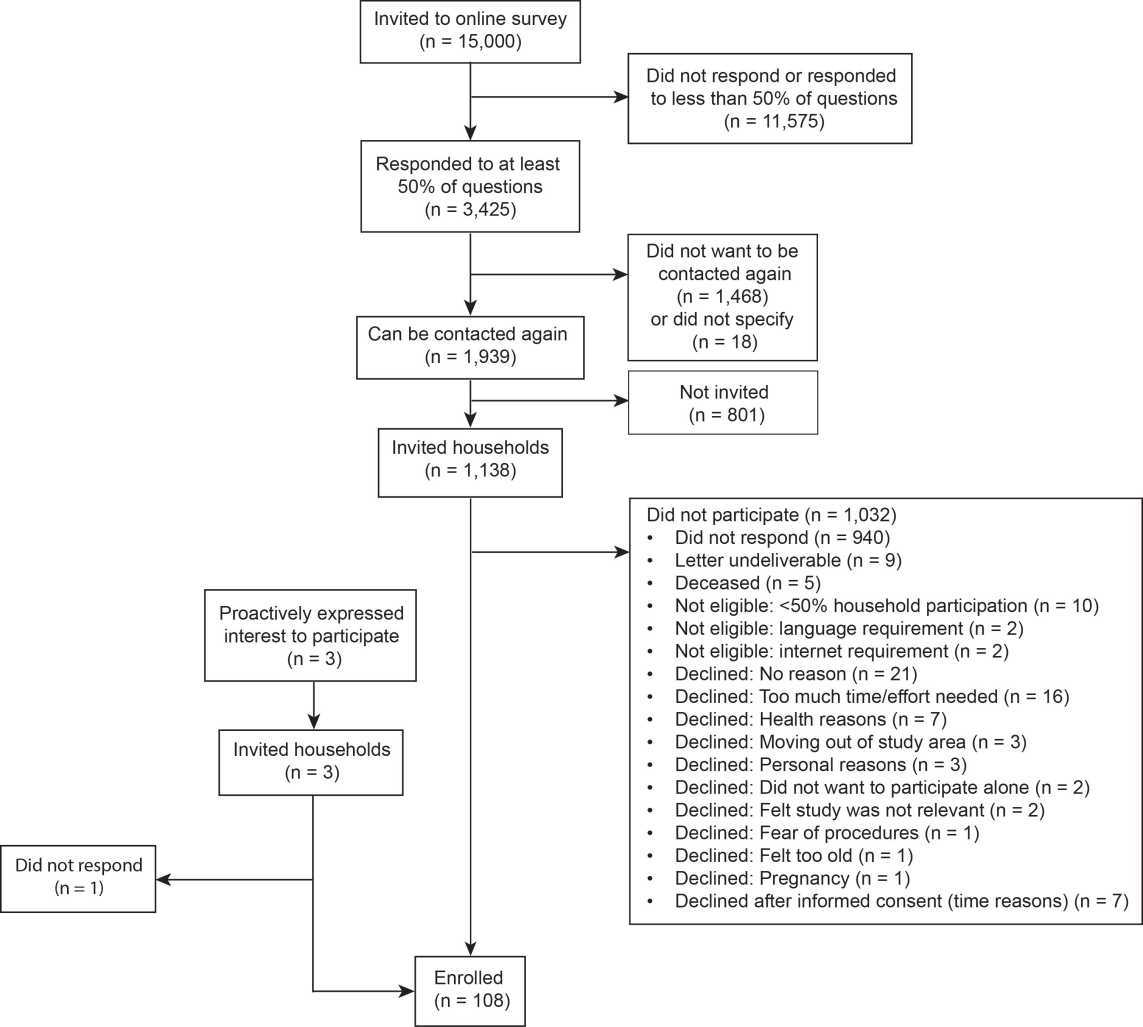
Flow diagram of study enrolment.

**Figure 2:**
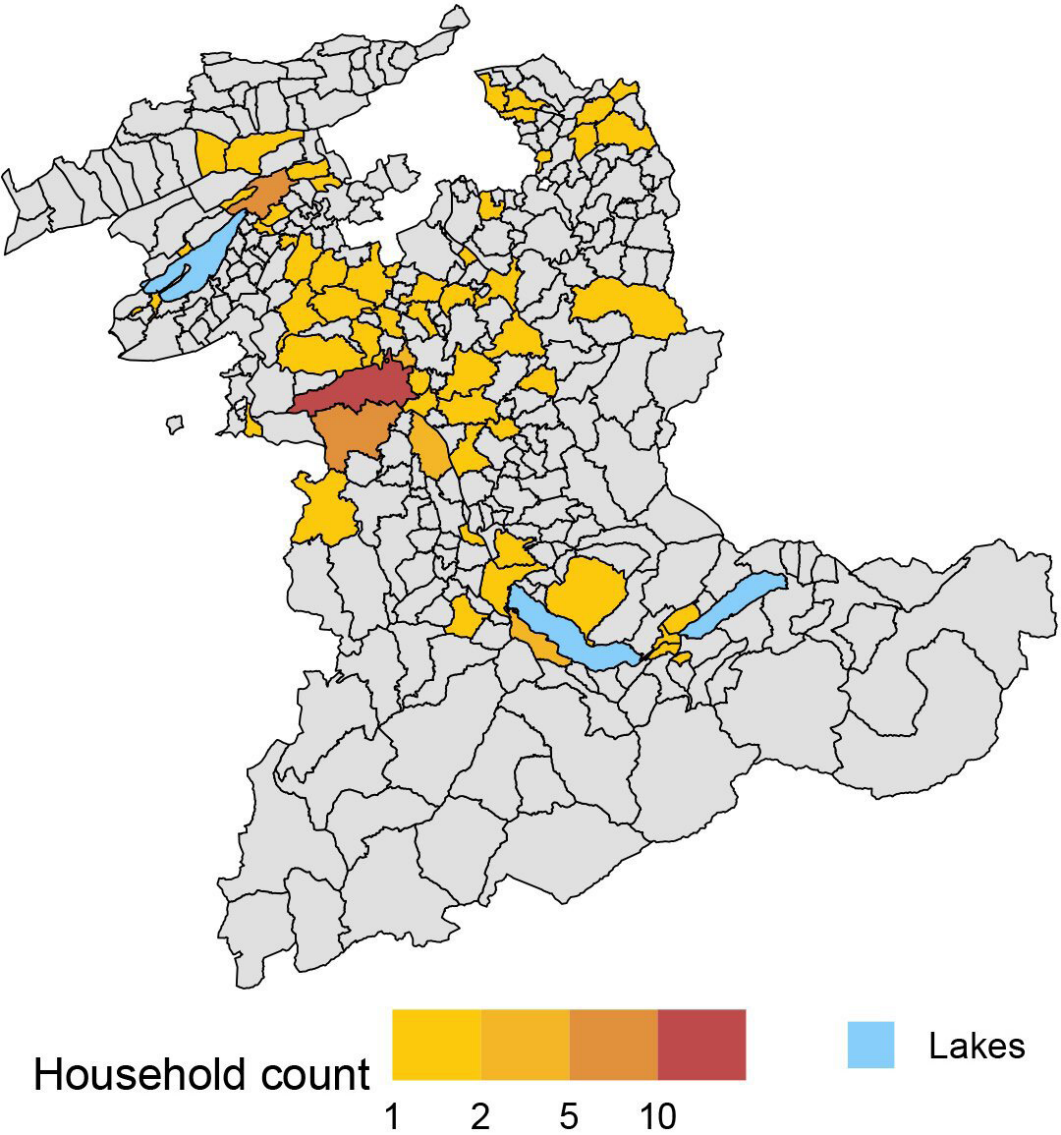
Distributions of households across the municipalities of the canton of Bern.

Between April and September 2023, we invited 1338 households, of which 106 (9%) agreed to participate (figure 1). We enrolled two additional households that had proactively contacted us wanting to participate, for a total of 108 households. Households came from 59 of the 338 municipalities in the canton of Bern (figure 2). The households included 193 people (ratio 1.8 persons per household, SD=1), among whom 32 were children and adolescents under the age of 18 (table 1). The median household size was 2 (IQR=1–2). There were 22/108 (20%) households with children, of which 16/108 (15%) agreed to include at least one child. There were 39/108 (36%, SD 4.6%) households with at least one cat (n=27), one dog (n=8) or one of each (n=4), of which 28/39 (72%, SD 7.2%) agreed to include at least one dog or cat, giving 28 households with 29 cats and 15 dogs. The median Swiss neighbourhood index of socioeconomic position of the included households was 65.3 (range 43.9–83.5). Five households chose to have their baseline visit at home. At the end of the follow-up, 101/108 (94%) households completed the study. One large household with pets withdrew consent due to personal reasons leading to lack of time, and six households were lost to follow-up despite three attempts to contact them (online supplemental table S4). 172 of 193 (89%) people completed the study, as did 22 owners of 34/44 (77%) pets. One cat died during the study period, and two cats were rehomed to a non-participating household outside the study area. The mean follow-up time for people to the last contact was 339 (95% CI 322 to 356, range 0–565) days and 335 (95% CI 299 to 370, range 3–489) days for pets.

**Table 1:** Baseline characteristics participants enrolled into the pilot study

Characteristics people	Included n=193 (%)	Total in household n=213 (%)
Adults	161 (92%*)	175
Children	32 (84%*)	38
Sex, n (%)		
Female	98 (51%)	
Male	90 (47%)	
No response	5 (2%)	
Age in years		
Median (IQR)	47 (29)	
Range	0–89	
Age categories in years		
0–17	32 (17%)	
18–29	11 (6%)	
30–64	105 (54%)	
65+	45 (23%)	
Household size, number of people (%)		
1	42 (22%)	
2	72 (37%)	
3	24 (13%)	
4	41 (21%)	
5+	14 (7%)	

Characteristics pets	Included n=44 (%)	Total in household n=69 (%)
Cats	29 (59%*)	49
Dogs	15 (75%*)	20

* Of total number of adults, children, cats or dogs living in participating household, respectively.

### Participant characteristics

The numbers of female and male participants were similar (table 1). The youngest participant was under 1 year old, and the oldest participant was aged 89 (median 47, IQR 29). Within households, 166/193 (86%) participants were from fully participating households (including 43 adults living alone).

The baseline characteristics of the enrolled participants were similar to the characteristics of the people we invited (online supplemental table S3).

In the univariable analysis, the odds of participation for people invited were lower in households with three or more people (OR 0.3, 95% CI 0.19 to 0.46) and higher for people with upper secondary or higher education (OR 2.83, 95% CI 1.15 to 9.37) and among women compared with men (OR 1.32, 95% CI 0.88 to 2.01). All variables from the univariable analysis were included into the multivariable model. The results for the adjusted odds in the multivariable analysis were similar (online supplemental table S3). We did not find evidence of strong associations between participation and age, sex, nationality or household location. All GVIFs were below 1.15, indicating no concerning multicollinearity.

189 of 193 (98%) people completed the baseline questionnaire, one person filled it in partially and three people did not fill it in. For the pets, owners completed the questionnaire for 43/44 (98%) pets and for one pet, the questionnaire was only partly completed. Of 161 adults/32 children, 158 (98.1%)/30 (93.8%) reported having ever received any vaccine. Of 125 (77.6%)/28 (87.5 %) with an immunisation card, 95 (76%)/24 (92.85%) provided a copy of it. For COVID-19, 152 (94.4%)/22 (68.8%) had received at least one dose of vaccine, >99% with an mRNA vaccine. In 82.8% of cats and 33.3% of dogs, a full vaccination record was available. Of those, 37.5% of cats and 100% of dogs were vaccinated according to current recommendations.^25^ All dogs and 18 of the 22 cats (81.8%) with outdoor access received regular antiparasitic treatment according to their owners. We found clinically relevant changes in the blood examination of 1/14 (7.1%) dogs and 11/28 (39.3%) cats. The dog had significant hyperglobulinaemia, and nine of the 11 cats had azotaemia. We recommended further investigation by the animal’s private veterinarian for all pets with significant changes. Pets lived in close contact with their owners, for example, 38 of 43 pets (88.4%) had access to the sofa and 26/43 (60.5%) were allowed in their owner’s bed.

### Participant satisfaction

After 1 month, 104/108 (96%) main household contact persons completed the questionnaire. Satisfaction was generally high. The study team identified the appointment booking procedure as an area for improvement in the main study. Two separate appointments for the consent discussion and the baseline visit were seen as unnecessary. Pet owners reported that the offer of the health check and blood test did not motivate them to enrol their pets.

### Biological sample collection

We obtained and stored at least one venous and/or capillary baseline blood sample from 184/193 (95%) people and 40/44 (91%) pets.

We received dried blood spot samples from 151 people out of 193 (78%) enrolled people and out of 172 (88%) people who completed the study. One sample reported could not be retrieved and was considered lost in the mail.

### Social contacts

**Figure 3:**
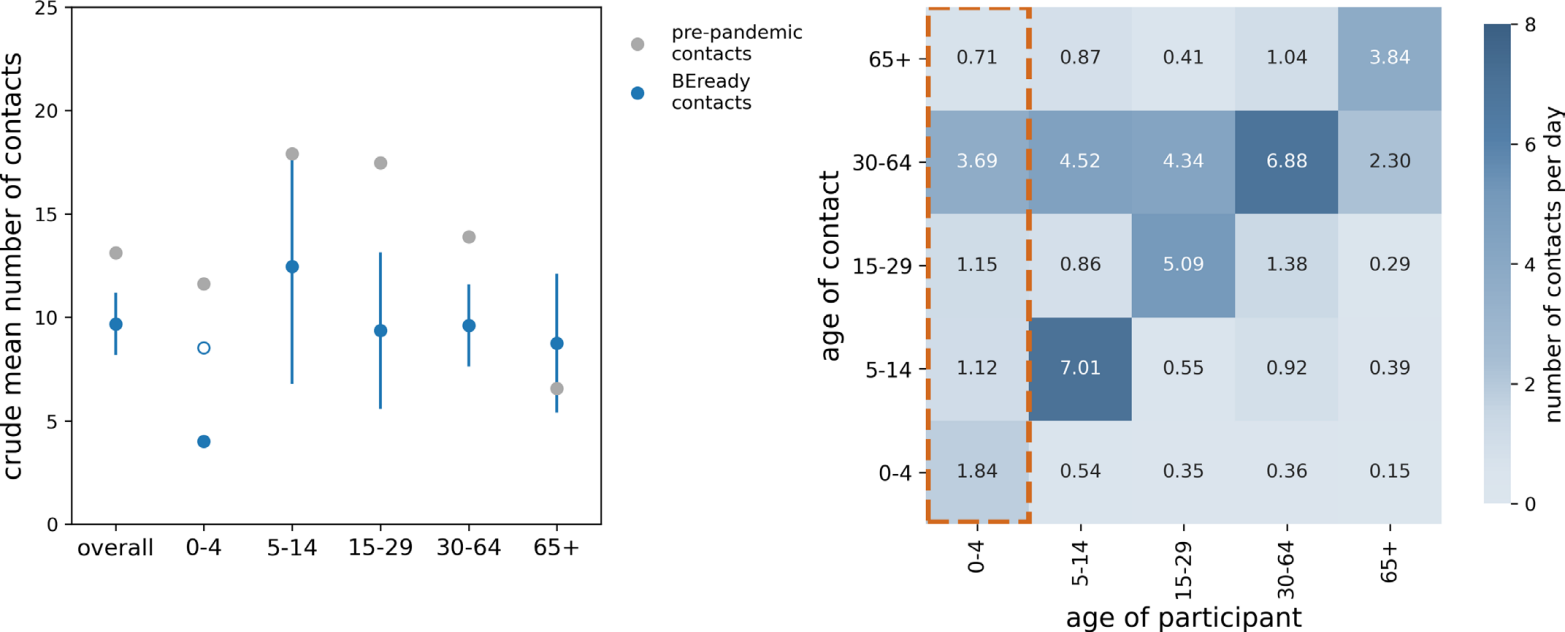
Analysis of social contacts. Left: crude mean number of contacts estimated from the survey data (in blue), overall and by age group. Symbols and error bars represent the sample mean and the associated 95% CI. Pre-pandemic values (in grey) are shown for comparison. The empty circle for the age group 0–4 years represents the imputed number of contacts, obtained by applying a scaling factor of 0.73 to pre-pandemic contacts. Right: estimated social contact matrix, adjusted by reciprocity and weighted by day of the week. Contacts for the age group 0–4 years (matrix column outlined in orange) were imputed using a scaled version of the pre-pandemic contacts.

The 193 participants enrolled in the study reported a total of 2063 contacts in a 24-hour period at baseline. After truncation of contacts at 50, the crude mean number of contacts was 9.7 (95% CI 8.2 to 11.2, figure 3). This corresponds to an overall 26% (95% CI 15% to 38%) reduction compared with pre-pandemic contacts. We estimated the mean number of contacts by age group to be 12.5 (6.8–18.1) for individuals aged 5–14 years, 9.4 (5.6–13.1) for 15–29 years, 9.6 (7.6–11.6) for 30–64 years, and 8.7 (5.4–12.1) for 65+ years (figure 3). A reduction in the number of contacts during the pandemic was observed in each age group, except for those aged 65+ years whose contacts were compatible with pre-pandemic levels (figure 3). Assuming a susceptibility and infectiousness profile by age for SARS-CoV-2, the ratio of the reproductive number R0 estimated from the empirical contacts and pre-pandemic contacts was 0.74 (0.61–0.89).

## Discussion

This pilot study enrolled 106/1138 (9%) of invited households; 193 people (1.8 per household) and 44 pets. After 1 year, 172/193 (89%) people in 101/108 (94%) of households completed a follow-up survey and 151/172 (88%) respondents returned a follow-up capillary blood sample. Numbers of contacts were 26% (95% CI 15% to 38%) lower than estimated pre-pandemic levels.^6 22^

### Strengths and limitations of the pilot study

A strength of this pilot study was the feasibility testing of all procedures from the invitation to participate, offering baseline assessment at a central study centre or at home, measurement of social contact patterns, assessment of retention at 1 year for humans and pets and symptom-driven nasal sampling in humans. The limited size of the pilot study means that there was low statistical power to investigate factors associated with participation. The pattern of responses was, however, similar to the findings of our larger online survey.^9^ Due to time and resource constraints, we used a convenience sample of previous survey respondents, as the primary objective was to evaluate the feasibility of study procedures and data collection methods rather than to produce population-representative estimates. To reduce the risk of selection bias in the main study, we will enrol a random sample of households, which is likely to result in even lower participation rates. The use of online questionnaires and mailing of self-collected samples was efficient and minimised direct contact between participants and study staff, which will be important during a pandemic. This approach does, however, limit the types and volumes of samples that can be collected. The baseline visit and online survey could be conducted in German or French, which are official languages in the canton of Bern. This can also be seen as a limitation because households with migration backgrounds and limited language capacity were unable to take part. A limitation of the pilot study and planned full cohort is the requirement for internet access, which excludes some older adults or those with unreliable internet access. Furthermore, the study included only cats and dogs, which are the most common household pets, but limits the range of animal-human infections which can be studied.

### Findings in relation to other population-based studies

The response rate in our pilot study was 9% among people invited at random from respondents to an online survey. The response rate of a new sample of households drawn at random from the canton of Bern may be too low to provide a sample that is representative of the population. We did not find other pandemic preparedness cohorts for comparison. In several cohort studies, which have enrolled individual participants from general population registers for different purposes since 2000, we also found low baseline response rates, from a pilot study for the Swiss Health Study to monitor the exposome (14%),^26^ to large national cohort studies in Europe, such as the UK Biobank of biomedical and genetic data (5.5%)^27^ or the *cohorte des CONSulTANts des Centre d’Examens de santé de la Sécurité sociale* (CONSTANCES) national cohort study (7.3%).^28^ A wide range of institutional, individual and study-related factors and larger societal dynamics influence participants’ decision to participate in health research.^29^ Many of these factors are beyond the control of researchers and our experience will not reverse the declines in participation in epidemiological studies observed over the past 40 years.^30^

### Meaning of the pilot study findings for the BEready cohort study

New pandemic preparedness cohort studies will be needed, even when the response rate is low. Many existing cohorts, which were not set up for the study of infectious diseases, were repurposed at the beginning of the COVID-19 pandemic.^31^,^34^ These studies were able to mobilise quickly for COVID-19 research but were not designed to study other infectious diseases. New studies can choose the age and type of study population, for example, individuals vs. households, and the data collected. As part of the pilot study, we collected data to estimate a social contact matrix. The preliminary analysis suggests that contact levels in Switzerland are still lower than estimates for the pre-pandemic period for most age groups. These data confirm the need for longitudinal study of contact patterns, which can be used in epidemiological and modelling studies as the ‘new normal’ before the next pandemic.

Household size was strongly associated with participation in our pilot study, with participants from larger households being less likely to take part than those in single person households. This aligns with findings from our online survey in the canton of Bern^9^ and from a Switzerland-wide online survey on willingness to participate in personalised health research performed in 2019–2020.^35^ Although we offered home visits to make scheduling easier for multiple household members, only five of 108 households accepted. We chose households as the unit of enrolment in BEready because of the need to understand secondary attack rates of directly transmitted pathogens. Fewer larger households mean that such studies will have less precision. The average household size in the canton of Bern is, however, only 2.15 so oversampling of large households and outreach to identify and engage people living in large households will be important. Infectious diseases are also associated with poverty and low socioeconomic position, of which overcrowding is a component.^36^ People in households of lower socioeconomic position and lower levels of education face multiple barriers to participating in clinical studies, for example, financial barriers such as missed work, lack of job flexibility, language barriers, low health literacy and distrust towards the healthcare system.^37^ In our pilot study, the odds of participation were higher among participants with higher incomes and higher levels of education, although CIs for these estimates were wide.

The enrolment of pets is a strength of the BEready cohort study, which allows a One Health perspective for the investigation of emerging pandemic threats. In the general population in Switzerland, 43% of households have pets, 30% cats and 12% dogs.^38^ In the pilot study, a similar proportion of all enrolled households had a cat or dog, but cat owners were less willing than dog owners (59% vs 75%) for their pet to take part. It is possible that this was because cats tend to become stressed when visiting veterinary clinics^39^ or that owners anticipate finding it harder to obtain samples at home. The offer of haematology and biochemistry analysis was not a motivation for participation. In the German National Cohort, investigators at two veterinary clinics examined the feasibility of teaching volunteer pet owners to mail home-collected samples from dogs and cats.^40^ They also found lower willingness to participate among cat owners than dog owners. They introduced home-sampling into the main cohort study to study zoonotic research questions, although they observed transport effects for stool samples. In the BEready pilot study, we provided videos for pet owners to refresh instructions about home-sampling. We have not identified other pandemic preparedness cohort studies that include both humans and pets. For the main BEready cohort study, we will collaborate with local veterinary practices to make baseline visits easier, especially for cat owners. We will also ask about close contact with other animals like horses, rabbits, rodents or turtles.

The completeness of collection of blood samples was high. We obtained and stored venous blood samples from 95% of people at the baseline visit and home-collected samples of dried blood spots from 88% of people at the end of the study. This high acceptance rate aligns with findings from the pilot study of the Swiss Health Study.^41^ We also stored samples from 91% of participating pets at baseline.

### Implications for a future pandemic preparedness cohort study

Our pilot study has implications for the conduct of cohort studies for pandemic preparedness research. Despite the low response rates, enrolling households into a population-based household cohort, with online questionnaires and self-sampling at follow-up, was feasible. Participant and community involvement in clinical studies has been shown to improve participation rates,^42^ and the Swiss Academy of Medical Sciences recommends fostering a culture of social responsibility for participation in clinical research.^43^ Some of our choices, such as the requirement for internet access, will facilitate research during a public health emergency but reduce access for some under-represented groups. For our main study, we have adapted the enrolment strategy and planned several activities to engage with the wider public in the canton of Bern, including those with lower levels of education, with community engagement activities to approach people directly and invite them to participate in exchange and all aspects of the research process. Strategies such as advertising opportunities on free media, using community events and facilitators, and partnering with local physicians are also planned.^37^ Based on experience from the pilot study and feedback from the questionnaire after 1 month, we have made changes to make it as easy as possible for households to join the study and to remain in it. We have implemented a new tool to allow people to book appointments directly online, outside working hours. To reduce the number of appointments, we have discontinued video conferences and will provide the study information in advance, online. We have also dropped the requirement for half, or more than half, of the people of the household to participate.

The timing of a new pandemic preparedness cohort study might also affect participation. The BEready study started after the emergency phase of the COVID-19 pandemic (sometimes referred to as ‘peacetimes’^44^). Cohorts which started during early waves of the pandemic might have benefited from heightened interest in the novel research topic, driven by the ongoing crisis, with response rates ranging from 11% to 43%.^45^,^48^ However, one disadvantage of studies launching only at the start of a pandemic is the lack of baseline (pre-pandemic) data and biological samples, which may be needed for comparison. One strength of our BEready study is that it will collect exactly that type of essential baseline data and samples, and that it will serve as a research platform for collaborations and data sharing with other researchers.

Establishing cohort studies is time-consuming, and speed is crucial when responding to a new pandemic. For BEready to be rapidly launched, it is essential to build the population’s trust; and community advisory panel and engagement activities are ongoing. In conclusion, the response rate to the pilot study shows that obtaining high levels of participant enrolment is challenging. The BEready pilot study shows that data collection without face-to-face contact with a study team is feasible for a household cohort study on pandemic preparedness and will be needed if control measures during a pandemic prevent in-person studies.

## Supplementary material

10.1136/bmjopen-2025-109555 online supplemental file 1

## Data Availability

Data are available upon reasonable request.
