# Supplementary material for: ‘Bern, get ready’, BEready, a household-based cohort study for pandemic preparedness research in Switzerland: pilot study
Source: BMJ Open. 2025 Dec 7;15(12):e109555. doi: 10.1136/bmjopen-2025-109555 (PMC12684152; doi:10.1136/bmjopen-2025-109555)
Supplement: online supplemental file 1 [file bmjopen-15-12-s001.pdf]

Supplemental material

'Bern, get ready', BEready, a household-based cohort study for pandemic preparedness research in Switzerland: pilot study

Eva Maria Hodel, Selina Wegmüller, Franziska Iff, Emily Lim, Karin Grimm, Lea Gasser, Laura Di Domenico, Simone Schuller, Gilles Wandeler, Nicola Low

Table of contents

Supplemental materials and methods 2

Selection of households to be invited 2

Follow-up at the end of study 2

Testing for respiratory viruses 2

Construction of social contact matrices 3

Supplemental tables and figures 4

Supplemental references 13

SUPPLEMENTAL MATERIALS AND METHODS

Selection of households to be invited

For our pilot study we invited respondents to the online survey [1], who had given permission to be contacted again. We oversampled larger households with children and pets as we knew from the online survey that they would be the more difficult to enrol, and we thought they were more likely to encounter feasibility issues. In each wave of invitations, we used a random sampler in R to select 150 households based on household size, presence of children in the household and pet ownership (supplemental figure S1). We aimed to invite equal numbers of households with one, two, three, four and five or more people, 75% of households with children, 20% of households with children aged under five years, and 50% of households with pets. To do this, each household was classified based on household size (i.e. one to five or more people), presence of children (yes/no), presence of children aged less than five years (yes/no), and ownership of pets (yes/no). For each of the possible combinations, we randomly selected a specific number of households for each wave of invitations. With an increasing number of waves, for certain combinations there were no more households left with these characteristics, and hence samples became gradually smaller than 150.

Follow-up at the end of study

The target follow-up time was 12±1 months. To allow sufficient time for participants to complete the online questionnaire, an automated invitation was sent out by REDCap to the participants 11 months after baseline. Up to five automated reminder emails were then sent at two-week intervals. The project management team made three attempts to contact any participant who had not opened the follow-up questionnaire by 13 months since the baseline visit: first by email, then by telephone or text message, and finally by surface mail. We classified people who still did not respond as “lost to follow-up” (supplemental table S4). We classified pets as either “lost to follow-up” in cases where the owner was also “lost to follow-up” or “other” in cases where the owner completed the study by filling in the follow-up questionnaire. The household was classified as “lost to follow-up” if all members were “lost to follow-up”.

Testing for respiratory viruses

The laboratory technician analysed nasal swabs by multiplex real-time polymerase chain reaction (Allplex RV Master Assay and Allplex Respiratory Panel 3, Seegene Inc., Seoul, Republic of Korea) for detection of severe acute respiratory syndrome

coronavirus 2 (SARS-CoV-2), human parainfluenza virus, influenza B virus, human adenovirus, influenza A virus, human metapneumovirus, human syncytial virus, human bocavirus 1/2/3/4, human rhinovirus A/B/C, human coronavirus 229E, human coronavirus NL63, and human coronavirus OC43 from nasopharyngeal swabs.

Construction of social contact matrices

First, we computed the crude mean number of contacts overall and stratified by age group. We truncated the number of contacts per participant to 50 to reduce the impact of outliers, as commonly done [2]. In our dataset, 5 out of 193 participants (2.6%) reported more than 50 contacts; specifically, they reported 59, 65, 74, 98 and 151 contacts, respectively. The data distribution is displayed in figure S1. Then, we compared the empirical estimates with pre-pandemic synthetic contacts reconstructed for Switzerland [2,3]. Secondly, we estimated a social contact matrix, adjusted for reciprocity of contacts and weighted by day of the week, using the 'socialmixr' package in R [4]. We generated 1,000 bootstrapped copies to account for uncertainty in the estimation of the contact matrix. We adjusted matrices for reciprocity using the population's age profile in the canton of Bern, provided by the Federal Statistics Office [5]. After applying the susceptibility and infectiousness profile by age group, we computed the largest eigenvalue of the empirical contact matrix, as this is proportional to the reproductive number R0 [6,7]. We used SARS-CoV-2 as an example. Hence, we set the infectiousness to one for all age groups and susceptibility to 0.5 and one for young individuals (<15 years old) and older individuals (15+ years old), respectively [2,7]. As the sample size for the youngest age group (under five years old) was too low to robustly estimate the number of contacts (only one participant), we imputed contacts for this age group using a scaled version of their corresponding pre-pandemic contacts. As a scaling factor, we took the ratio of the largest eigenvalue of the empirical matrix and the pre-pandemic matrix, considering only the matrix elements for the other age groups (five or more years old), as done in other works [8].

SUPPLEMENTAL TABLES AND FIGURES

Table S1. Biological samples collected.

Species	Time point	Sample type	Sampling material used	Volume	Aliquotes stored	Storage temperature
Human	Baseline	Venous blood	S-Monovette EDTA K3E, cap red, SARSTEDT AG, Sevelen, Switzerland	7.5 mL	7 × 500 µL plasma<br>1 × 500 µL buffy coat	-80°C
			S-Monovette Serum Gel CAT, SARSTEDT AG, Sevelen, Switzerland	7.5 mL	7 × 500 µL serum	-80°C
		Capillary blood	Whatman protein saver card, Cytiva, Marlborough, United States of America		5 dried blood spots	-80°C
Cat	Baseline	Venous blood	Micro sample tube EDTA K3E, 1.3 ml, SARSTEDT AG, Sevelen, Switzerland	≤3 mL	≤3 × 500 µL plasma<br>≤1 × 500 µL buffy coat	-80°C
			Sample tube, Serum Gel CAT 2.7 ml, SARSTEDT AG, Sevelen, Switzerland		≤3 × 500 µL serum	-80°C
Dog	Baseline	Venous blood	Micro sample tube EDTA K3E, 1.3 ml, SARSTEDT AG, Sevelen, Switzerland	≤5 mL	≤3 × 500 µL plasma<br>≤1 × 500 µL buffy coat	-80°C
			Sample tube, Serum Gel CAT 2.7 ml, SARSTEDT AG, Sevelen, Switzerland		≤3 × 500 µL serum	
Human	Disease event	Nasal swab	• FLOQSwabs regular flocked swabs (502CS01) in adults and older children<br>• FLOQSwabs minitip flocked swabs (516CS01) in children less than 3 years old, Copan Italia S.p.A., Brescia, Italy<br>• UTM, Copan Italia S.p.A., Brescia, Italy	3 mL	2 × 1.5 mL	-80°C
Cat	Disease event	Pharyngeal swab	• FLOQSwabs minitip flocked swabs (516CS01), Copan Italia S.p.A., Brescia, Italy<br>• UTM, Copan Italia S.p.A., Brescia, Italy	3 mL	2 × 1.5 mL	-80°C
Dog	Disease event	Nasal swab	• FLOQSwabs minitip flocked swabs (516CS01), Copan Italia S.p.A., Brescia, Italy<br>• Universal transport medium: UTM, Copan Italia S.p.A., Brescia, Italy	3 mL	2 × 1.5 mL	-80°C
Human	One year follow-up	Capillary blood	Whatman protein saver card, Cytiva, Marlborough, United States of America		5 dried blood spots	-80°C

Table S2. Source of questions used from previous studies.

**Questions in BEready**	**Timing***	**Source**	**Description of source**
Medical history and travel history	BL, FU	Swiss Health Study pilot phase [9]	The **Swiss Health Study** is to be conducted at a national level. It aims to investigate the influence of the environment, chemicals, lifestyle, infectious diseases and personal characteristics on health and the most common widespread diseases. In 2019-2021, methods that are to be used in the large national study were trialled in a pilot phase in 1,349 adults, and the necessary infrastructure was developed and tested.
Vaccination against COVID-19 (adapted)	BL	CoMix[10]	**CoMix** is a study that followed households across 17 European countries in real-time during the COVID-19 pandemic. The survey asked people about their awareness, attitudes and behaviours in response to COVID-19 and measured how these changed over time.
Smoking, alcohol, and illicit drugs (adapted)	BL	Swiss Health Survey 2017 [11]	The **Swiss Health Survey** has been conducted every five years since 1992. The data is collected via a telephone, followed by a written questionnaire. The permanent resident population aged 15 and over in private households is surveyed. The survey covers health status and health-related behaviour.
Social contacts	BL, FU	CoMix questionnaires [10]	See above
Age group for the social contacts	BL, FU	Sentinella reporting system[12]	Since 1986, the **Sentinella reporting system** has monitored common, non-reportable communicable diseases such as influenza, pertussis (whooping cough) and mumps in Switzerland. General practitioners from all over Switzerland report cases of illness anonymously to the Federal Public Health Office.
Closeness of contact between pet owners and their pets	BL, FU	Two previous studies[13,14]	Joosten *et al.* conducted an online survey among pet owners in Belgium to study the closeness of interactions between dogs and their owners. Dazio *et al*. studied the acquisition and carriage of multidrug-resistant organisms in dogs and cats presented to small animal practices and clinics in Switzerland.
Occupation, social status, and place of residence	BL, FU	Swiss Structural Survey 2022 [15]	The **Swiss structural survey** is a component of the population census and complements information obtained from registers with additional statistics on the structure of the population. The structural survey is conducted annually and samples at least 200,000 persons aged 15 years and older, living in a private household in Switzerland. It collects information on population, households, families, housing, employment, mobility, education, language and religion.
		Socio-Professional Categories 2021 [16]	**Socio-professional categories** are a classification system for mapping the social structure of the population. The categories are based on various basic variables for the employed people (occupational status, occupation practised, and the highest level of education completed). For the other, only a distinction is made between apprentices, the unemployed and the economically inactive persons.

		Regional classifications [17]	The Federal Statistical Office has developed a method for statistically mapping Switzerland's current urban structures by defining "areas with an urban character". Both morphological and functional criteria are considered for this **regional classification**. Each municipality is assigned to a category based on standardised criteria and threshold values.
		Swiss Household Panel [18] wave 22	The **Swiss Household Panel** (SHP) is a unique longitudinal survey in the social sciences interviewing all household members of a random sample of private households in Switzerland since 1999. The principal aim of this annual panel study is to observe social change, in particular the dynamics of changing living conditions in the population of Switzerland.
Quality of life and health status	BL, FU	In adults and teenagers from 14 years: EQ-5D-5L [19]	**EQ-5D** is a standardised measure of health-related quality of life developed by the EuroQol Group to provide a simple, generic questionnaire for use in clinical - and economic appraisal and population health surveys. EQ-5D assesses health status in terms of five dimensions of health and is considered a 'generic' questionnaire because these dimensions are not specific to any one patient group or health condition.
		Children aged 3 years and less than 14 years with a parent or caregiver responding as proxy: EQ-5D-Y [20]	
Respiratory symptoms (adapted)	DE	Bern Basel Infant Lung Development cohort [21]	The **Bern Basel Infant Lung Development** (BILD) cohort was established in 1999 to study the effects of genetics and the environment on infant and child lung growth and development in relation to lung diseases such as asthma.
	DE	InfluenzaNet [22]	**InfluenzaNet** is a network of volunteers in 12 European countries that is self-reporting their health status weekly through real-time syndromic surveillance of influenza and COVID-19.
Gender roles	FU	COGEN [23]	**COvid and GENder** (COGEN) is an observational cohort study in Switzerland that investigated if non-biological aspects of being male or female (e.g. social roles and personality traits), the so-called "gender" dimension, may be associated with observed sex imbalance in COVID-19 outcomes.
Polarisation	FU	Published study [24]	This study looked at affective polarization, i.e. the emotional attachment to in-group members and hostility toward out-group members, regarding the COVID-19 vaccination in six European countries. They used an 11-point scale, ranging from 0 "I completely reject them" to 10 "I completely support them", to rate people's opinions about the COVID-19 vaccination. They also asked people to rate their feelings toward two groups: (a) people who get vaccinated (b) people who do not get vaccinated. Respondents were asked to describe their feelings using a thermometer scale ranging from -5 to +5, with -5 meaning they feel very cold and negative toward the group and +5 means they feel very sympathetic and positive toward the group.
Marginality	FU	Several concepts [25–30]	The questions were derived from different concepts of marginality to cover socio-cultural, economic and systemic dimensions of marginality in a 4-point Likert scale.

* BL: at baseline; DE: during disease events; FU: at one year follow-up.

Table S3. Agreement to participate according to demographic and socio-economic variables of the household member who received the invitation letter.

	Pilot Study		Participation in pilot study*						Online survey	
	**Included**	**Invited**	**Univariable models**			**Multivariable model**			**Responders**	**Invited**
**Characteristic of invited person**	**n = 106**	**n = 1,138**	**OR**	**CI**	***p***	**OR**	**CI**	***p* of LRT**	**n = 3,425**	**n = 15,000**
**Age**, years**, median (IQR)	55 (42–67)	48 (38–61)	1.02	1.00 –1.03	0.014	1.00	0.99–1.02	0.601	49 (37–62)	46 (34–60)
**Sex****								0.095		
Male	42	515	1.00 (ref.)			1 (ref.)			1,626	7,352
Female	64	623	1.32	0.88–2.01	0.180	1.43	0.94–2.21		1,799	7,648
**Nationality**								0.194		
Swiss (incl. dual nationals)	79	861	1.00 (ref.)			1 (ref.)			2,574	Unavailable
Non-Swiss	18	206	0.94	0.54–1.58	0.830	1.58	0.86–2.80		643	Unavailable
No response or prefer not to say	8	66	1.36	0.58–2.79	0.438	1.71	0.71–3.67		208	Unavailable
**Education**								0.046		
Compulsory or less	4	107	1.00 (ref.)			1 (ref.)			488	Unavailable
Upper secondary or tertiary	100	1,009	2.83	1.15–9.37	0.046	3.10	1.23–10.41		2,856	Unavailable
Other or no response	2	22	2.71	0.36–14.94	0.269	3.28	0.42–18.84		81	Unavailable
**Household size****								<0.001		
1	36	229	1.00 (ref.)			1 (ref.)			651	3,000 / 185,820****
2	41	300							885	3,000 / 170,629****
3	11	191	0.30	0.19–0.46	<0.001	0.28	0.16–0.45		723	3,000 / 54,814****
4	12	288							811	3,000 / 53,981****
5+	6	130							355	3,000 / 24,729****
**Urban-rural typology**/*****								0.497		
Urban	64	635	1.00 (ref.)			1 (ref.)			1,881	8141
Intermediary or rural	42	499	0.80	0.53–1.20	0.290	0.86	0.56–1.32		1,530	6808
**Income** [CHF]								0.599		
< 3’000	2	43	1.00 (ref.)			1 (ref.)			193	Unavailable
3’000 – 9’000	62	579	2.43	0.72–15.13	0.228	2.44	0.70–15.47		1,693	Unavailable
9’000	30	380	1.78	0.51–11.27	0.440	2.21	0.60–14.39		916	Unavailable
Don’t know, no response or prefer not to say	12	136	2.00	0.52–13.20	0.377	2.16	0.54–14.49		623	Unavailable

Abbreviation: CI = 95% confidence interval, IQR = inter-quartile range, LRT = likelihood ratio test, OR = odds ratio, *p* = p-value.

***** Observations for uni- and multivariable analysis n = 1129.

** As provided by Amt für Informatik und Organisation des Kantons Bern (KAIO) on 07.09.2022.

*** Federal Statistical Office typology of municipalities 2012 [17]. Not available for municipality numbers 334, 624, 876, 881 and 993 as per 01.01.2024.

**** Number of households invited / total number of households canton of Bern on the day the sample was drawn

Table S4. Status of households and participants at end of study.

Participant and outcome	Questionnaire	Questionnaire completed						
**Main contact person in household (n = 108)**	**Individual**	**Yes**			**Part.**	**No**		
	**Household**	**Yes**	**Part.**	**No**	**Yes**	**Yes**	**Part.**	**No**
Completed		90	1	2	3	3	1	
Lost to follow-up								7
Withdrew consent						1		
**Other people in household (n = 85)**	**Individual**	**Yes**			**Part.**	**No**		
Completed		71			1			
Lost to follow-up						11		
Withdrew consent						2		
**Pets (n = 44)**	**Individual**	**Yes**			**Part.**	**No**		
Completed		34						
Died						1		
Left study area						2		
Lost to follow-up						1		
Other reason for non-completion*						4		
Withdrew consent					1	1		

* Pet owner completed own questionnaire but did not complete pet's questionnaire. Part. = Partially

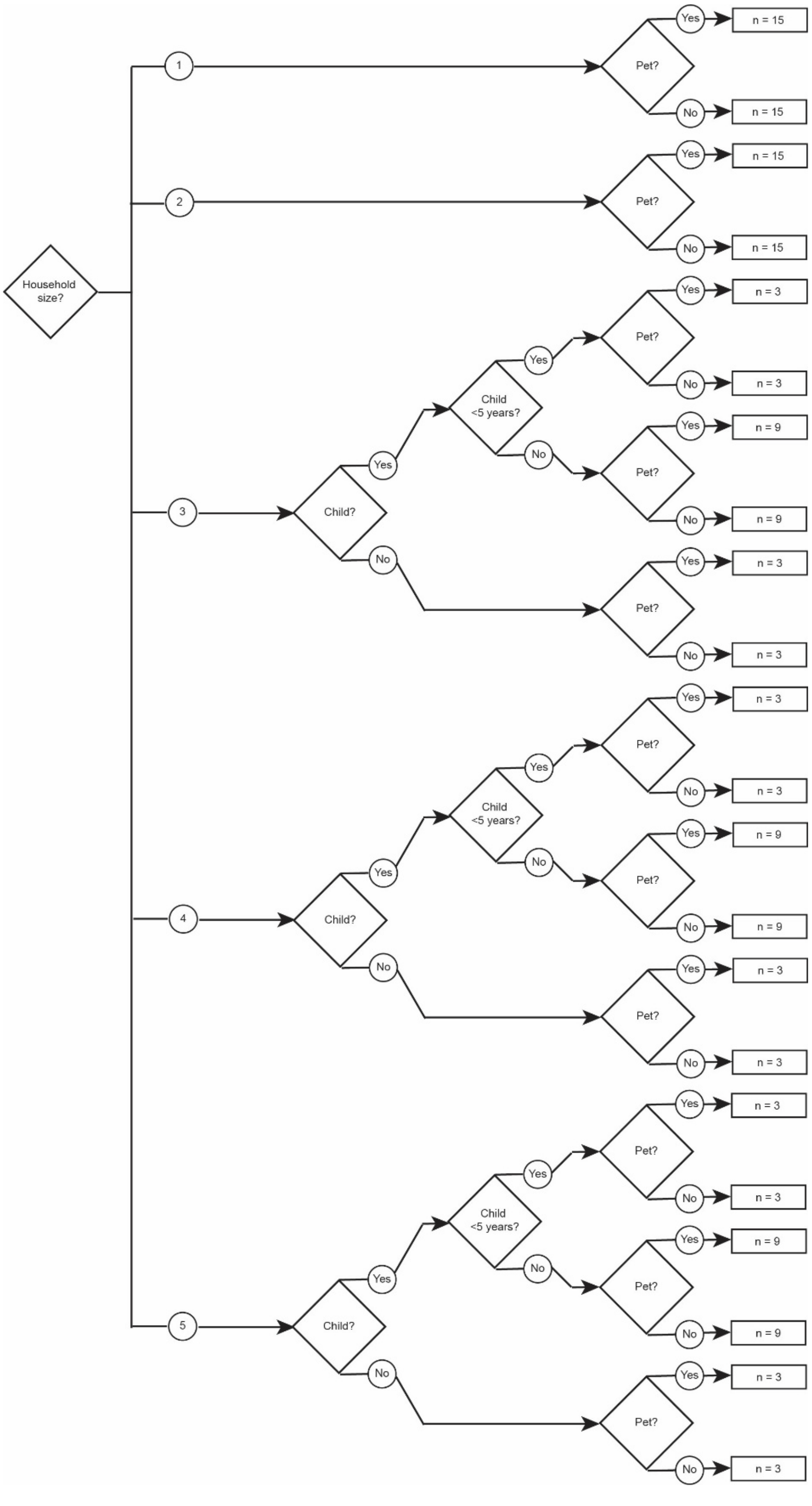

Figure S1. Distribution of randomly selected households in each stratum.

In each wave of invitations, we used a random sampler in R to select 150 households based on household size, presence of children in the household and pet ownership. With an increasing number of waves, certain strata could no longer be completely filled and hence samples became gradually smaller than 150.

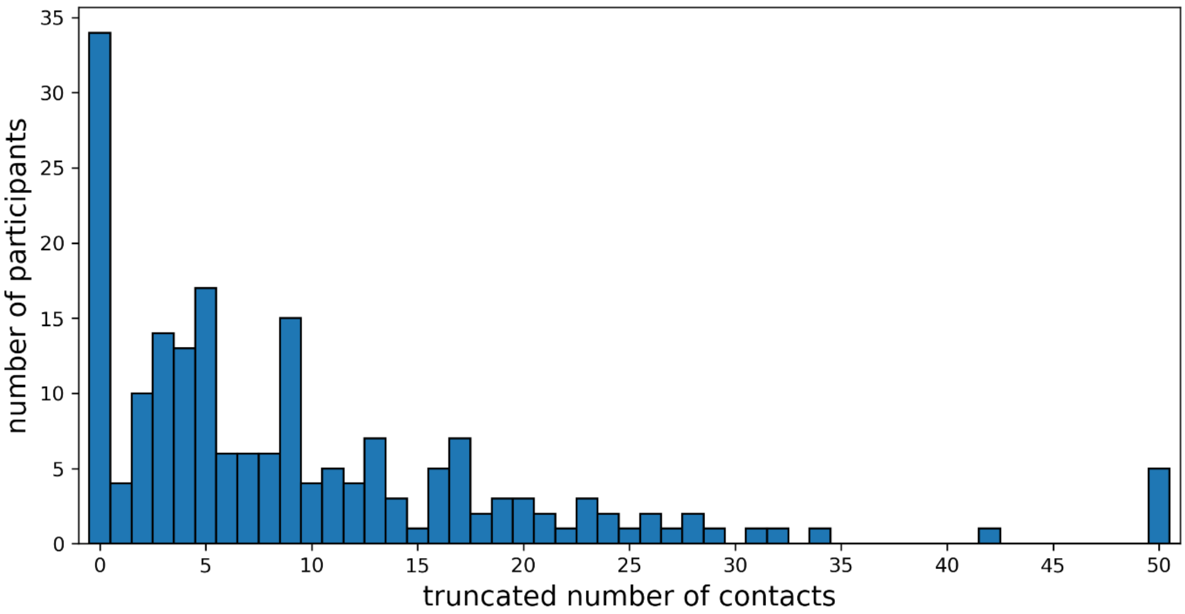

Figure S2. Histogram of the number of contacts per participant, truncated at 50 contacts.

SUPPLEMENTAL REFERENCES

1 Ipekci AM, Hodel EM, Filsinger M, *et al.* Who would take part in a pandemic preparedness cohort study? The role of vaccine-related affective polarisation: cross-sectional survey. *medRxiv* 2025.04.26.25326349. doi: https://doi.org/10.1101/2025.04.26.25326349

2 Reichmuth ML, Heron L, Beutels P, *et al.* Social contacts in Switzerland during the COVID-19 pandemic: Insights from the CoMix study. *Epidemics*. 2024;47:100771. doi: 10.1016/j.epidem.2024.100771

3 Prem K, Zandvoort K van, Klepac P, *et al.* Projecting contact matrices in 177 geographical regions: An update and comparison with empirical data for the COVID-19 era. *PLoS Comput Biol*. 2021;17:e1009098-.

4 Funk S, Willem L, Gruson H, *et al.* Socialmixr: Social Mixing Matrices for Infectious Disease Modelling.

5 Federal Statistical Office. Population. 2024. https://www.bfs.admin.ch/bfs/en/home/statistiken/bevoelkerung.html (accessed 20 February 2024)

6 Mossong J, Hens N, Jit M, *et al.* Social Contacts and Mixing Patterns Relevant to the Spread of Infectious Diseases. *PLoS Med*. 2008;5:e74. doi: 10.1371/journal.pmed.0050074

7 Munday JD, Jarvis CI, Gimma A, *et al.* Estimating the impact of reopening schools on the reproduction number of SARS-CoV-2 in England, using weekly contact survey data. *BMC Med*. 2021;19:233. doi: 10.1186/s12916-021-02107-0

8 Jarvis CI, Van Zandvoort K, Gimma A, *et al.* Quantifying the impact of physical distance measures on the transmission of COVID-19 in the UK. *BMC Med*. 2020;18:124. doi: 10.1186/s12916-020-01597-8

9 Morand Bourqui R, Nusslé SG, von Goetz N, *et al.* Towards a Swiss health study with human biomonitoring: Learnings from the pilot phase about participation and design. *PLoS One*. 2023;18:e0289181. doi: 10.1371/journal.pone.0289181

10 Reichmuth ML, Heron L, Riou J, *et al.* Socio-demographic characteristics associated with COVID-19 vaccination uptake in Switzerland: longitudinal analysis of the CoMix study. *BMC Public Health*. 2023;23:1523. doi: 10.1186/s12889-023-16405-0

11 Federal Statistical Office. Schweizerische Gesundheitsbefragung 2017. Übersicht. Neuchâtel 2018.

12 The Sentinella reporting system in Switzerland. https://www.sentinella.ch

13 Joosten P, Van Cleven A, Sarrazin S, *et al.* Dogs and Their Owners Have Frequent and Intensive Contact. *Int J Environ Res Public Health*. 2020;17:4300. doi: 10.3390/ijerph17124300

14 Dazio V, Nigg A, Schmidt JS, *et al.* Acquisition and carriage of multidrug-resistant organisms in dogs and cats presented to small animal practices and clinics in Switzerland. *J Vet Intern Med*. 2021;35:970–9. doi: 10.1111/jvim.16038

15 Federal Statistical Office. Structural Survey 2022. 2022.

16 Federal Statistical Office. Sozioprofessionelle Kategorien (SPK) 2021-Operationalisierung der sozioprofessionellen Kategorien. 2021.

17 Federal Statistical Office. Raumgliederungen der Schweiz. 2012.

18 Tillmann R, Voorpostel M, Antal E, *et al.* The Swiss Household Panel (SHP). *Jahrb Natl Okon Stat*. 2022;242:403–20. doi: 10.1515/jbnst-2021-0039

19 Herdman M, Gudex C, Lloyd A, *et al.* Development and preliminary testing of the new five-level version of EQ-5D (EQ-5D-5L). *Quality of Life Research*. 2011;20:1727–36. doi: 10.1007/s11136-011-9903-x

20 Wille N, Badia X, Bonsel G, *et al.* Development of the EQ-5D-Y: a child-friendly version of the EQ-5D. *Quality of Life Research*. 2010;19:875–86. doi: 10.1007/s11136-010-9648-y

21 Fuchs O, Latzin P, Kuehni CE, *et al.* Cohort Profile: The Bern Infant Lung Development Cohort. *Int J Epidemiol*. 2012;41:366–76. doi: 10.1093/ije/dyq239

22 Paolotti D, Carnahan A, Colizza V, *et al.* Web-based participatory surveillance of infectious diseases: the Influenzanet participatory surveillance experience. *Clinical Microbiology and Infection*. 2014;20:17–21. doi: 10.1111/1469-0691.12477

23 Gebhard CE, Hamouda N, Gebert P, *et al.* Sex versus gender-related characteristics: which predicts clinical outcomes of acute COVID-19? on behalf of the COGEN Investigators. *Intensive Care Med*. 2022;48:1652–5. doi: 10.1007/s00134-022-06836-5

24 Filsinger M, Freitag M. Asymmetric affective polarization regarding COVID-19 vaccination in six European countries. Published Online First: 2024. doi: 10.1038/s41598-024-66756-w

25 Dennis RM. Marginality. The Wiley Blackwell encyclopedia of race, ethnicity, and nationalism. 2016;V:1–4.

26 Mehretu A, Pigozzi BWm, Sommers LM. Concepts in social and spatial marginality. *Geogr Ann Ser B*. 2000;82:89–101. doi: 10.1111/j.0435-3684.2000.00076.x

27 Wacquant LJD. The Rise of Advanced Marginality: Notes on its Nature and Implications. *Acta Sociologica*. 1996;39:121–39. doi: 10.1177/000169939603900201

28 Baah FO, Teitelman AM, Riegel B. Marginalization: Conceptualizing patient vulnerabilities in the framework of social determinants of health—An integrative review. *Nurs Inq*. 2019;26. doi: 10.1111/nin.12268

29 von Braun J, Gatzweiler FW, editors. *Marginality*. Dordrecht: Springer Netherlands 2014.

30 Déry S, Leimgruber W, Zsilincsar W. Understanding Marginality: Recent Insights from a Geographical Perspective. *Hrvatski geografski glasnik/Croatian Geographical Bulletin*. 2012;74:5–18. doi: 10.21861/HGG.2012.74.01.01
