# Supplementary material for: ‘Bern, get ready’, BEready, a household-based cohort study for pandemic preparedness research in Switzerland: pilot study
Source: BMJ Open. 2025 Dec 7;15(12):e109555. doi: 10.1136/bmjopen-2025-109555 (PMC12684152; doi:10.1136/bmjopen-2025-109555)
Supplement: Supplementary file 2 [file bmjopen-2025-109555.reviewer_comments.pdf]

PEER REVIEW HISTORY

BMJ Open publishes all reviews undertaken for accepted manuscripts. Reviewers are asked to complete a checklist review form and are provided with free text boxes to elaborate on their assessment. These free text comments are reproduced below.

ARTICLE DETAILS

Title (Provisional)

"Bern, get ready", BEready, a household-based cohort study for pandemic preparedness research in Switzerland: pilot study

Authors

Hodel, Eva Maria; Wegmüller, Selina; Iff, Franziska; Lim, Emily; Grimm, Karin; Gasser, Lea; Di Domenico, Laura; Schuller, Simone; Wandeler, Gilles; Low, Nicola

---

VERSION 1 - REVIEW

**Reviewer**	**1**
**Name**	**Courvoisier, Delphine**
**Affiliation**	**Geneva University Hospitals, Division of clinical epidemiology**
**Date**	**15-Sep-2025**
**COI**	**None**

This article presents the results of a pilot cohort testing with the goal to assess the feasibility of having a cohort allowing to assess pandemic response and supply data for emerging infectious diseases. This cohort includes household and some pets.

Overall, the article is well-detailed and clear, and these initiatives are important to describe to facilitate the creation of other cohorts and also to communicate about available sources of data in case of emerging infectious diseases. Nevertheless, a few points would improve the usefulness of this article.

Major points:

- This article mainly presents a study design and information about the data collected. In line with the WHO call to share data, these data should be available to be shared. While the authors do indicate that they "are available upon reasonable request", previous studies have shown that this is often insufficient. The authors should clarify criteria that allow data to be obtained, timeframe before requests are answered, as well as potential costs or conditions to get the data (e.g., it has to be a collaboration) to really encourage researchers to apply for these collaborations.

- the authors should also discuss in the text that they aim, or not, to share these data (not only in the data sharing statement). An example of such practice is the ARGOS register (doi: 10.1136/bmjopen-2021-048946)

- methods: if I understand correctly, the first analysis is at the household level. If correct, please clarify how you adjust for individual characteristics (e.g., line 290: age, sex, nationality, education) since the statistical unit is the household. Is it the person of reference? Alternatively, if this is at the individual level, a linear mixed-effect model should be used (or GEE).

- Results, social contacts, line 365: the results on the R0 estimated are not sufficiently described, in methods or in results. Obtaining this number requires a lot of assumptions which are unknown here. The authors should either delete this sentence, which would be my preference, or describe in details how they obtain it, without only referring to other publications.

- Figure 3 (left panel): this figure would be more informative with an overlay of all individual points. Also, the number of truncated contact values should be at least indicated. In real life, these may have been superspreader events and are thus quite worthy of interest.

**Reviewer**	**2**
**Name**	**Azizmohammad Looha, Mehdi**
**Affiliation**	**Shahid Beheshti University of Medical Sciences**
**Date**	**20-Sep-2025**
**COI**	**None**

This manuscript addresses an important and timely question by piloting a novel household-based cohort (BEready) for pandemic preparedness, integrating both human and pet participation within a One Health framework. The study is valuable in demonstrating the feasibility of remote data and sample collection, long-term follow-up, and high retention. However, the work is limited by a very low enrolment rate, potential selection bias from the recruitment strategy, under-reporting of certain outcomes (e.g., social contacts), and several issues with clarity, formatting, and reporting standards. Overall, while the study provides useful insights, substantial revisions are required to strengthen methodological transparency, improve data presentation, and ensure consistency with reporting guidelines. Therefore, I recommend major revision rather than rejection.

General Comments

Major

The manuscript's sample selection poses a major concern. Recruiting only households with prior online survey participation and internet access introduces selection bias, as internet-

based cohorts are “particularly prone to selection bias” [1]. Please discuss how this may have affected the generalizability of the findings.

The study is described as adhering to various reporting checklists, but the rationale for this choice is not entirely clear. For example, the TIDieR (Template for Intervention Description and Replication) checklist was developed for describing interventions [2]. Since BEready is presented as an observational pilot study rather than an interventional one, it is not obvious why TIDieR would apply here.

Using the STROBE checklist is appropriate for a cohort study, but it’s important that all items are fully addressed. If any are missing, it becomes difficult to properly assess the study’s strengths and weaknesses.

The introduction’s aim (“investigate the potential response”) is vague. It would be better to specify what is meant by “potential response” (presumably, the expected participation rate or willingness to join) and list specific feasibility outcomes.

Results section should explicitly report the findings from the social contact survey (e.g. average contacts by age group), since this was highlighted as an outcome.

Title

The title’s punctuation is awkward. Using quotes and a comma inside (“Bern, get ready”) is nonstandard.

Abstract

Major

Minor

Some sentences in the abstract and text are long and should be tightened.

Introduction

Major

The research gap is described only in general terms (e.g., lack of timely data, lack of social contact data), but it is not clearly explained why establishing a new cohort in Bern is necessary when other European cohorts already exist. The manuscript should also clarify what distinguishes BEready from existing initiatives (e.g., UK Biobank or other COVID-related cohorts) and how it adds unique value.

Minor

The term “potential response” in this context is unclear. If it means expected participation rate, it would be better to say “expected participation rate” or “anticipated enrolment.”

Why a household (vs. individual) cohort is valuable.

Methods

Major

The citation of TIDieR is unnecessary for this observational study. TIDieR is meant for describing interventions. If no intervention was tested, remove this reference to avoid confusion. (The STROBE citation for cohort studies is appropriate.)

The entire Methods section should be structured in accordance with the STROBE guidelines for cohort studies. Unless the authors can provide a compelling justification for deviating from STROBE, the manuscript should follow this framework. This guideline has several items including study design, setting, participants, variables, data source/measurements, bias, study size, statistical analysis.

Authors invited only respondents from a prior online survey, rather than a true random sample of all households. This limitation should be acknowledged in Methods or Discussion.

The criterion "half or more of household members agreed" is noted. Explain what happens if exactly half the members agree, or if only one adult agrees. Also justify why this threshold (50%) was chosen.

Please summarize handling of missing data and follow-up losses.

The description of the multivariable regression analysis is insufficient. It is unclear on what basis variables were selected for inclusion in the models. The manuscript should specify: (i) which variables were considered, (ii) the criteria for their inclusion (e.g., based on prior knowledge, univariate significance, or stepwise procedures), and (iii) whether multicollinearity was assessed. Without this information, the transparency and reproducibility of the analysis are limited.

Minor

How many households expressed interest but were deemed ineligible?

Please define primary and secondary outcomes.

Results

Major

The results on recruitment and retention are clearly presented. It may help to break these into sub-sections (e.g. "Recruitment", "Baseline participation", "Follow-up") to guide the reader.

Minor

The social contact survey results are not detailed in the Results text.

Please summarize the main findings quantitatively (e.g., average contacts by age group).

Provide summary statistics (mean or median) for household size, since the analysis found household size to be important.

footnote " Of total in household" is somewhat confusing.

It is important to report the univariate and multivariate impact of varaibles on outcome. And show the method for inclusion of variables in multivariate analysis.

Six households were lost and one withdrew. If known, briefly mention any reasons for loss (e.g. relocation, withdrew because of pets, etc.)

Discussion

Major

One important limitation that is not clearly stated is the potential for selection bias. Because participants were recruited only from an online panel and were required to have internet access, certain groups such as households without internet or those who never responded to the prior survey were automatically excluded. This should be acknowledged as a limitation that affects the study's external validity.

Minor

The discussion on pet inclusion is insightful. If space permits, it would be helpful for the authors to indicate whether any findings (even qualitative observations) on animal health were made, or to state clearly that such analyses will be reported separately. At present, the focus is solely on participation rates among pets.

1. Hatch, E.E., et al., Evaluation of Selection Bias in an Internet-based Study of Pregnancy Planners. Epidemiology, 2016. 27(1).

2. Hoffmann, T.C., et al., Better reporting of interventions: template for intervention description and replication (TIDieR) checklist and guide. Bmj, 2014. 348: p. g1687.

VERSION 1 - AUTHOR RESPONSE

#	Reviewer's comment	Authors' response	Location
	**Reviewer 1** Dr. Delphine Courvoisier		
	This article presents the results of a pilot cohort testing with the goal to assess the feasibility of having a cohort allowing to assess pandemic response and supply data for emerging infectious diseases. This cohort includes household and some pets.<br><br>Overall, the article is well-detailed and clear, and these initiatives are important to describe to facilitate the creation of other cohorts and		

	also to communicate about available sources of data in case of emerging infectious diseases. Nevertheless, a few points would improve the usefulness of this article.		
	Major points:		
R1.1	This article mainly presents a study design and information about the data collected. In line with the WHO call to share data, these data should be available to be shared. While the authors do indicate that they "are available upon reasonable request", previous studies have shown that this is often insufficient. The authors should clarify criteria that allow data to be obtained, timeframe before requests are answered, as well as potential costs or conditions to get the data (e.g., it has to be a collaboration) to really encourage researchers to apply for these collaborations.	Thank you for your comment. We have expanded our data sharing statement with the explanation why data is not publicly available: "The data from this study are not publicly available because they contain coded personal data and are therefore subject to legal obligations. Coded means that all reference data that could be used to directly identify participants are stored separately and can only be linked with the data and samples by using the randomly generated identification number. Data and/or samples are available upon reasonable request from the investigators. " We have also added the link to our request form and clarified that "Fees may apply for data and sample preparation and transfer, depending on the nature of the collaboration."	Data sharing statement, lines 631–650
R1.2	the authors should also discuss in the text that they aim, or not, to share these data (not only in the data sharing statement). An example of such practice is the ARGOS register (doi: 10.1136/bmjopen-2021-048946)	Thank you for directing us to the very helpful profile of your ARGOS registry. We specified that BEready will serve as a research platform in the discussion, i.e. "One strength of our BEready study is that it will collect exactly that type of essential baseline data and samples, and that it will serve as a research platform for collaborations and data sharing with other researchers."	Discussion, Implications for a future pandemic preparedness cohort study, lines 583–585
R1.3	methods: if I understand correctly, the first analysis is at the household level. If correct, please clarify how you adjust for individual characteristics (e.g., line 290: age, sex, nationality, education) since the statistical unit is the	We apologise for any confusion. We now clarify "In this pilot study, each household nominated a main contact person, typically the person who received the invitation letter. The main contact person registered the household online using Research Electronic Data Capture (REDCap) tools hosted at	Materials and methods, Study setting and participants, lines 192–196<br><br>Materials and methods,

	household. Is it the person of reference? Alternatively, if this is at the individual level, a linear mixed-effect model should be used (or GEE).	the University of Bern [14,15].", early in the Methods.<br><br>We have also split the sentence to clarify that the person receiving the invitation letter is the unit of analysis. Lastly, we have added the following sentence. "<u>The unit of analysis was the person receiving the invitation letter. For households that did not accept the invitation to participate in the pilot study, we only had demographic and socio-economic information for the invited person, not for other household members.</u>"<br><br>As there is only one person per household, there is no household clustering effect, and we believe that a logistic regression model is more appropriate than a linear mixed-effect model or generalised estimating equations.	Statistical analyses, lines 306, 310–313
R1.4	Results, social contacts, line 365: the results on the R0 estimated are not sufficiently described, in methods or in results. Obtaining this number requires a lot of assumptions which are unknown here. The authors should either delete this sentence, which would be my preference, or describe in details how they obtain it, without only referring to other publications.	We apologise for the omission of an important sentence about R0. The list of assumptions behind the estimate of R0 was fully reported in the Supplementary Material of our original submission. We have now included the following sentence in the Methods section to clarify this: "<u>We used the estimated contact matrices to quantify the variation in the reproductive number R0 compared to pre-pandemic contacts. Details on the construction of the contact matrix and derivation of R0 are reported in the supplementary material.</u>"	Materials and methods: Statistical analyses, lines 336–339
R1.5	Figure 3 (left panel): this figure would be more informative with an overlay of all individual points. Also, the number of truncated contact values should be at least indicated. In real life, these may have been superspreader events and are thus quite worthy of interest.	The quantity displayed in figure 3 (left panel) is the mean number of contacts (point estimate and associated uncertainty). For completeness, we now present the distribution of the number of contacts per participant in figure S2 of the supplementary material. "<u>In our dataset, 5 out of 193 participants (2.6%) reported more</u>	Supplementary materials: Construction of social contact matrices, lines 52–54, and figure S2, lines 98–99

		than 50 contacts; specifically, they reported 59, 65, 74, 98 and 151 contacts, respectively." This is now specified in the text in the supplementary material.<br><br>We acknowledge that truncating high contact numbers may overlook contact patterns that could result in a superspreading event. This is a very well-known limitation of the social contact matrix approach, which focuses on the average number of contacts, without explicitly accounting for individual heterogeneity. The social contact matrix approach still allows us to draw meaningful insights into age-stratified contact patterns.	
	**Reviewer 2** Dr. Mehdi Azizmohammad Looha		
	This manuscript addresses an important and timely question by piloting a novel household-based cohort (BEready) for pandemic preparedness, integrating both human and pet participation within a One Health framework. The study is valuable in demonstrating the feasibility of remote data and sample collection, long-term follow-up, and high retention. However, the work is limited by a very low enrolment rate, potential selection bias from the recruitment strategy, under-reporting of certain outcomes (e.g., social contacts), and several issues with clarity, formatting, and reporting standards. Overall, while the study provides useful insights, substantial revisions are required to strengthen methodological transparency, improve data presentation, and ensure		

	consistency with reporting guidelines. Therefore, I recommend major revision rather than rejection.		
	General Comments<br>Major		
R2.1	The manuscript's sample selection poses a major concern. Recruiting only households with prior online survey participation and internet access introduces selection bias, as internet-based cohorts are "particularly prone to selection bias" [1]. Please discuss how this may have affected the generalizability of the findings.	We agree that there is a risk of selection bias. We mentioned the selection bias and the justification for the convenience sample in the discussion. We would like to reassure the reviewer that we will use a random sample for the main cohort study. We now mention this: "<u>Due to time and resource constraints, we used a convenience sample of previous survey respondents, as the primary objective was to evaluate the feasibility of study procedures and data collection methods rather than to produce population-representative estimates. To reduce the risk of selection bias in the main study, we will enrol a random sample of households, which is likely to result in even lower participation rates.</u>"	Discussion, Strengths and limitations of the pilot study, lines 463–468
R2.2	The study is described as adhering to various reporting checklists, but the rationale for this choice is not entirely clear. For example, the TIDieR (Template for Intervention Description and Replication) checklist was developed for describing interventions [2]. Since BEready is presented as an observational pilot study rather than an interventional one, it is not obvious why TIDieR would apply here.<br>Using the STROBE checklist is appropriate for a cohort study, but it's important that all items are fully addressed. If any are missing, it becomes difficult to properly assess the study's strengths and weaknesses.	We found the TIDieR guideline helpful to make sure that our study processes could be replicated. To avoid confusion, we have removed the mention of TIDieR at the start of "Materials and methods".<br><br>We have followed the STROBE guidelines as diligently as possible and provided the checklist with our initial submission, indicating for each item on the checklist where it can be found in the manuscript. An updated checklist, reflecting the page numbers of our revised manuscript, has been uploaded.	Materials and methods, lines 175–176<br><br>STROBE checklist

R2.3	The introduction's aim ("investigate the potential response") is vague. It would be better to specify what is meant by "potential response" (presumably, the expected participation rate or willingness to join) and list specific feasibility outcomes.	We indeed mean the "expected participation rate" and have adapted the sentence to: "The objective of this study was to investigate the expected participation rate and to test the feasibility of procedures for a population-based household cohort study for pandemic preparedness research in the canton of Bern."	Introduction, line 167
R2.4	Results section should explicitly report the findings from the social contact survey (e.g. average contacts by age group), since this was highlighted as an outcome.	Thank you for this request. We now report quantitative findings about the average number of contacts by age group in the results section, i.e. "We estimated the mean number of contacts by age group to be 12.5 (6.8–18.1) for individuals aged 5-14 years, 9.4 (5.6–13.1) for 15–29 years, 9.6 (7.6–11.6) for 30-64 years, and 8.7 (5.4–12.1) for 65+ years (figure 3). A reduction in the number of contacts during the pandemic was observed in each age group, except for those aged 65+ years whose contacts were compatible with pre-pandemic levels (figure 3)."	Results, Social contacts, lines 439–443
R2.5	Title<br>The title's punctuation is awkward. Using quotes and a comma inside ("Bern, get ready") is nonstandard.	"Bern, get ready" is a direct address to Bern (which ultimately refers to the people of the canton of Bern in Switzerland) and therefore requires a comma to separate the people or place being spoken to from the rest of the sentence. We have adapted the title to address comment E.1.	
R2.6	Abstract<br>Major<br><br>Minor<br>Some sentences in the abstract and text are long and should be tightened.	We have shortened the sentences in the Objectives and Conclusions parts of the abstract.	Abstract, lines 73, 75 and 100–103
R2.7	Introduction<br>Major<br>The research gap is described only in general terms (e.g., lack of timely data, lack of social contact data), but it is not clearly explained why	We have extended the introduction by stating that current initiatives have not been established with pandemic preparedness in mind; hence, the type of data they collect may not encompass all relevant aspects. The added section reads	Introduction, lines 138–142

	establishing a new cohort in Bern is necessary when other European cohorts already exist. The manuscript should also clarify what distinguishes BEready from existing initiatives (e.g., UK Biobank or other COVID-related cohorts) and how it adds unique value.	as follows: "Active longitudinal cohort studies are valuable in pandemic research because they collect rich clinical and laboratory data over time, enabling deeper insight, for instance, into effect modifiers of disease outcomes [4]. To our knowledge, none of the existing large population-based longitudinal cohorts has been designed to specifically collect longitudinal data relevant for pandemic preparedness."	
R2.8	Minor<br>The term "potential response" in this context is unclear. If it means expected participation rate, it would be better to say "expected participation rate" or "anticipated enrolment."	See R2.3 above.	
R2.9	Why a household (vs. individual) cohort is valuable.	We expanded the section to provide a more detailed rationale, i.e. "Infections typically cluster within households due to shared environmental and behavioural exposures and the potential for intra-household transmission of pathogens which spread through direct contact. Households, therefore, are the unit of enrolment to allow the study of both primary and secondary infection events, to facilitate the collection of shared exposure data, and to reduce bias arising from treating related individuals as independent units."	Introduction, lines 147–153
R2.10	Methods<br>Major<br>The citation of TIDieR is unnecessary for this observational study. TIDieR is meant for describing interventions. If no intervention was tested, remove this reference to avoid confusion. (The STROBE citation for cohort studies is appropriate.)<br>The entire Methods section should be structured in	We addressed the comment about TIDierR in our response to comment R2.2.<br><br>We used the STROBE statement and ensured that we addressed all items. To keep the manuscript concise, we sometimes combined more than one item under a single heading.	

	accordance with the STROBE guidelines for cohort studies. Unless the authors can provide a compelling justification for deviating from STROBE, the manuscript should follow this framework. This guideline has several items including study design, setting, participants, variables, data source/measurements, bias, study size, statistical analysis.		
R2.11	Authors invited only respondents from a prior online survey, rather than a true random sample of all households. This limitation should be acknowledged in Methods or Discussion.	See R2.1 above.	
R2.12	The criterion "half or more of household members agreed" is noted. Explain what happens if exactly half the members agree, or if only one adult agrees. Also justify why this threshold (50%) was chosen.	We have clarified the statement as "half, or more than half," throughout the manuscript to show that a household was eligible if exactly half of the members agreed.<br><br>We have added the justification for doing so, i.e. "If fewer than half of the household members agreed to participate, the study nurse did not offer a baseline visit for enrolment. In this pilot study, we aimed to test	Materials and methods, Study setting and participants, line 197<br><br>Materials and methods, Study procedures, line 212<br><br>Discussion, Implications for a future pandemic preparedness cohort study, lines 573–574<br><br>Materials and methods, Study procedures, lines 215–219

		procedures for data and sample collection among participants experiencing symptoms of respiratory infections in households with at least two people."	
R2.13	Please summarize handling of missing data and follow-up losses.	We specified in the methods that "We excluded observations with missing values" in the logistic regression models.	Materials and methods, Statistical analyses, line 316
R2.14	The description of the multivariable regression analysis is insufficient. It is unclear on what basis variables were selected for inclusion in the models. The manuscript should specify: (i) which variables were considered, (ii) the criteria for their inclusion (e.g., based on prior knowledge, univariate significance, or stepwise procedures), and (iii) whether multicollinearity was assessed. Without this information, the transparency and reproducibility of the analysis are limited.	We have provided the following additional detail in the text:<br><br>(i) for the variables considered and (ii) the criteria for inclusion, "We selected age, education, household size, urban-rural typology and income as potential confounders based on the analysis of our online survey [9]. The online survey showed that willingness to participate was less likely among older participants and those in larger households, and more likely among participants with the highest educational level, near the city of Bern than in rural areas, and those with the highest income. The additional confounders, sex and nationality, were selected based on subject matter knowledge."<br><br>(ii) We already specified in the methods that "We performed multivariable analyses to adjust for potential confounders and expressed the results as adjusted odds ratios (aOR, with 95% CI). Potential confounders (age, sex, nationality, education, household size, urban-rural typology, and income) were selected **a priori**." and we therefore did not make any further changes.<br><br>(iii) We also specified that "We assessed multicollinearity by calculating generalised variance inflation factors (GVIFs) using the	Materials and methods, Statistical analyses, lines 320–327<br><br>Materials and methods, Statistical analyses, lines 329–331.<br><br>Results, Participant characteristics, line 404.

		VIF() function in the regclass package in R." and "All GVIFs were below 1.15, indicating no concerning multicollinearity."	
R2.15	Minor<br>How many households expressed interest but were deemed ineligible?	We reported reasons for ineligibility in the flow chart (figure 1) under "Not eligible". Fourteen households were excluded: ten because fewer than half of their members agreed to participate, two due to insufficient language skills, and two because they lacked internet access. We did not add this to the text.	Figure 1
R2.16	Please define primary and secondary outcomes.	In the methods, we stated "The primary outcome was enrolment as a percentage, which we calculated by dividing the number of households enrolled by the number of households invited for the pilot study." We now specify that other outcomes were secondary outcome measures: "Secondary outcomes included the associations between the agreement to participate and the demographic and socio-economic variables of the invited household member, the number of social contacts, the proportion of participants from whom samples were collected, the proportion of participants with completed questionnaires, and the proportion of participants responding after 12 months."	Materials and methods, Statistical analyses, lines 300–304
R2.17	Results<br>Major<br>The results on recruitment and retention are clearly presented. It may help to break these into sub-sections (e.g. "Recruitment", "Baseline participation", "Follow-up") to guide the reader.	To maintain the order of results in accordance with the STROBE guidelines, we introduced the following additional subheadings and rearranged bits of text where necessary: "Participant numbers", "Participant characteristics", "Participant satisfaction", and "Biological sample collection".	Results, lines 359, 384, 422, 429
R2.18	Minor<br>The social contact survey results are not detailed in the Results text.	See R2.4 above.	

	Please summarize the main findings quantitatively (e.g., average contacts by age group).		
R2.19	Provide summary statistics (mean or median) for household size, since the analysis found household size to be important.	We added this information to the results section, saying "The median household size was 2 (IQR = 1–2)."	Results, Participant numbers, line 366
R2.20	footnote " Of total in household" is somewhat confusing.	We specified that the percentages are based on the "total number of adults, children, cats or dogs living in participating household, respectively".	Results, Table 1, line 391
R2.21	It is important to report the univariate and multivariate impact of varaibles on outcome. And show the method for inclusion of variables in multivariate analysis.	The detailed results, which include both our univariate and multivariate estimates of odds ratios, can be found in supplementary Table S3. We clarified in the text that the results of both analyses were similar and referred to supplementary table S3. We also clarified that "All variables from the univariable analysis were included into the multivariable model".<br><br>Regarding the method for inclusion of variables in the multivariate analysis, see R1.14 above.	Results, Participant characteristics, lines 400–402
R2.22	Six households were lost and one withdrew. If known, briefly mention any reasons for loss (e.g. relocation, withdrew because of pets, etc.)	We have provided more details on the household that withdrew consent. Unfortunately, we have no further details from the households that were lost to follow-up as they did not respond to our three attempts to contact them (email, phone/text message, and letter sent by surface mail). To clarify we have added more details to the sentence on the households that did not complete. "One large household with pets withdrew consent due to personal reasons leading to lack of time, and 6 households were lost to follow-up despite three attempts to contact them (supplementary Table S4)."	Results, Participant numbers, lines 376–378
R2.23	Discussion<br>Major<br>One important limitation that	The requirement for internet access has been mentioned under "Strengths and limitations of the	

	is not clearly stated is the potential for selection bias. Because participants were recruited only from an online panel and were required to have internet access, certain groups such as households without internet or those who never responded to the prior survey were automatically excluded. This should be acknowledged as a limitation that affects the study's external validity.	pilot study", i.e. "A limitation of the pilot study and planned full cohort is the requirement for internet access, which excludes some older adults or those with unreliable internet access." We have not made additions to the text.<br><br>For the selection bias see also R2.1 above.	
R2.24	Minor<br>The discussion on pet inclusion is insightful. If space permits, it would be helpful for the authors to indicate whether any findings (even qualitative observations) on animal health were made, or to state clearly that such analyses will be reported separately. At present, the focus is solely on participation rates among pets.	We have added more details about the baseline characteristics of the pets, e.g. "All dogs and 18 of the 22 cats (81.8%) with outdoor access received regular antiparasitic treatment according to their owners. We found clinically relevant changes in the blood examination of 1/14 (7.1%) dogs and 11/28 (39.3%) cats. The dog had significant hyperglobulinemia, and nine of the eleven cats had azotemia. We recommended further investigation by the animal's private veterinarian for all pets with significant changes. Pets lived in close contact with their owners, for example, 38 of 43 pets (88.4%) had access to the sofa and 26/43 (60.5%) were allowed in their owner's bed."	Results, Participant characteristics, lines 414–421
	1. Hatch, E.E., et al., Evaluation of Selection Bias in an Internet-based Study of Pregnancy Planners. Epidemiology, 2016. 27(1).<br>2. Hoffmann, T.C., et al., Better reporting of interventions: template for intervention description and replication (TIDieR) checklist and guide. Bmj, 2014. 348: p. g1687.		

VERSION 2 - REVIEW

**Reviewer**	**1**
**Name**	**Courvoisier, Delphine**
**Affiliation**	**Geneva University Hospitals, Division of clinical epidemiology**
**Date**	**31-Oct-2025**
**COI**	

I thank the reviewers for their thorough answers to my comments.
